# The expression of PD-L1 in salivary gland carcinomas

**DOI:** 10.1038/s41598-019-49215-9

**Published:** 2019-09-04

**Authors:** Domenic Vital, Kristian Ikenberg, Holger Moch, Matthias Rössle, Gerhard F. Huber

**Affiliations:** 1 Department of Otorhinolaryngology, Head and Neck Surgery, Zurich University Hospital and University of Zurich, Frauenklinikstrasse 24, CH-8091 Zurich, Switzerland; 2 Department of Pathology and Molecular Pathology, Zurich University Hospital and University of Zurich, Schmelzbergstrasse 12, CH-8091 Zurich, Switzerland; 3 Department of Otorhinolaryngology, Head and Neck Surgery, Kantonsspital St. Gallen, Rorschacher Strasse 95, CH-9007 St. Gallen, Switzerland; 4 Department of Pathology, Luzerner Kantonsspital, Spitalstrasse, CH-6000 Lucerne 16, Switzerland

**Keywords:** Head and neck cancer, Surgical oncology

## Abstract

Objective was to analyze the role of PD-L1 and its relation to demographic, patho-clinical and outcome parameters in salivary gland carcinoma (SGC) patients. Patients treated for salivary gland carcinomas between 1994 and 2010 were included. A retrospective chart review for baseline characteristics, pathohistological, clinical and outcome data was performed. Immunohistochemistry for PD-L1 was performed using tissue microarrays. PD-L1 expression was assessed in tumor cells and tumor-infiltrating immune cells (TIIC) and statistical analysis with regard to baseline and outcome data was performed. Expression of PD-L1 (by means ≥1% of the cells with PD-L1 positivity) was present in the salivary gland carcinoma cells of 17%, in the TIIC of 20% and in both tumor cells and TIIC of 10% the patients. PD-L1 expression in tumor cells and both tumor cells and TIIC was related to tumor grading (p = 0.035 and p = 0.031, respectively). A trend towards higher grading was also seen for PD-L1 expression in TIICs (p = 0.058). Patients with salivary duct carcinomas and PD-L1 expressing TIICs showed a significantly worse DFS and OS (p = 0.022 and p = 0.003, respectively), those with both tumor cells and TIIC expressing PD-L1 a significantly worse DFS (p = 0.030). PD-L1 expression is present in 17% and 20% of salivary gland carcinoma cells and TIIC. Ten percent of the patient showed a PD-L1 positivity in both tumor cells and TIIC. This is related to high tumor grading and therefore might be a negative prognostic factor.

## Introduction

Salivary gland carcinoma (SGC) is a rare disease with an incidence of 25–30 per one million individuals, accounting for about 0.5% of all malignancies and 5% of all head and neck cancers^1,2^. Regardless of its histological subtype, curative treatment consists of a surgical resection with or without postoperative adjuvant radiation therapy^1,3,4^, although adjuvant treatment options like (chemo)radiation do not have a detectable impact on survival in highly aggressive subtypes like salivary duct carcinomas^5^. There is also no standard treatment of proven efficacy for patients with unresectable primaries/recurrences or patients with distant metastasis^1^. The benefit of chemotherapies like combinations of cisplatin, doxorubicin and cyclophosphamide was reported to be minimal with a prognosis that remains poor^1,6–8^.

Immunotherapies have shown remarkable success in various entities not limited to non-small cell lung cancer and malignant melanoma. All these therapies share a similar mode of action directing the body’s own immune system to dispose tumor cells^9,10^. Although the immune system is one of the major defense elements in antitumor response, tumor cells derive from the patient’s own cells and therefore maintain many natural autoimmune defense mechanisms that can prevent tumor immune destruction^11^. The programmed death (PD)-1/programmed death ligand 1 (PD-L1) pathway is one of these mechanisms to elude antitumor immune response, by which expansion and activity of cytotoxic T cells is suppressed by binding of the tumor cell’s PD-L1 on the PD-1 receptor on tumor-specific T cells^1,12,13^. A targeted immune therapy with PD-1/PD-L1 inhibitors (e.g. pembrolizumab, atezolizumab) can disrupt these pathways and enhance the immune system’s antitumor activity^10^.

So far, not much is known about the role of PD-L1 in SGC. Nevertheless, it often affects younger patients and efficacy of chemotherapeutic agents is limited as mentioned above, why it would be of advantage to identify factors predicting outcome with regard to a patient-tailored treatment. Up to present, there were only a few studies analyzing the role of PD-L1 in SGC, considering the immune profiling of PD-L1 and –L2 in adenoid-cystic carcinomas (ACC) or examining the correlation between PD-L1 expression and clinic-pathologic behavior of SGC^1,6,14^. Therefore, objective of our study was to analyze the role of PD-L1 and its relation to demographic, patho-clinical and outcome parameters in our cohort of patients with SGC.

## Materials and Methods

### Patients

The study protocol was approved by the ethics committee of the local authority (Kantonale Ethikkommission Zürich (KEK); KEK-ZH-Nr. 2010-0206/0). All experiments and analyses were performed in accordance with the relevant guidelines and regulations. Informed consent was obtained. Patient with salivary gland carcinomas, who underwent treatment between January 1^st^, 1994 and December 31^st^, 2010 (17 years) were eligible to be included in this study. The same cohort was used in a previous study focusing on the expression of cancer testis antigens in salivary gland carcinomas^15^. Patients with incomplete data sets or insufficient tissue quality or quantity were excluded. Retrospective chart review focused on age at initial diagnosis, sex, tumor entity and localization, TNM classification (UICC, 7^th^ ed.), tumor grading, resection margins, extranodal extension of lymph node metastasis, perineural invasion, blood and lymphatic vessel invasion, recurrence and survival. Given that there is no universally used grading system for the different SGC entities, the grading system according to Brandwein *et al*.^16^ was used for mucoepidermoid carcinoma (MEC) and the grading system according to Szanto *et al*.^17^ for acinic cell carcinoma (AcCC). Grading of all the other SGC entities was bases on similarity to normal tissue of origin, grade of pleomorphism/anaplasia, vascular and perineural invasion and mitotic activity.

All patients underwent resection of their SGC, frequently treated with postoperative radiation therapy. Radiation therapy was indicated in case of large primaries with infiltration of surrounding tissues, high-grade histology, perineural or lymphovascular invasion, close or positive resection margins, multiple lymph node metastasis or lymph node metastasis with extranodal extension. None of the patients underwent treatment with PD-1/PD-L1 inhibitors.

Disease free survival (DFS) is the period of time without evidence of disease (i.e. persistence, recurrence or metastasis) after primary treatment. Disease-specific survival (DSS) and overall survival (OS) were defined as the duration after primary treatment to death due to SGC or any other reason, respectively. Oncologic follow-up was performed at the Department of Otorhinolaryngology, Head and Neck Surgery, University Hospital of Zurich. It consisted of clinical examinations and routine use of ultrasonography as well as cross-sectional imaging (MRI and/or PET-CT). Minimal oncologic standard surveillance time in SGC patients with salivary gland carcinomas is 10 years.

### Tissue microarray construction/immunohistochemistry

**Figure 1:**
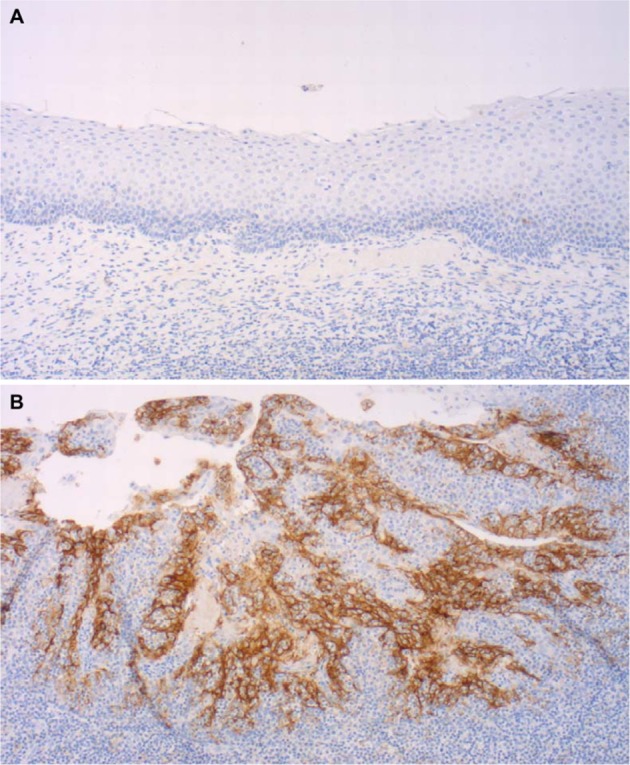
Tonsil tissue stained for PD-L1 demonstrating the quality of the applied staining protocol with clearly negative superficial squamous epithelium (**A**) and strong diffuse staining in reticular crypt epithelial cells (**B**), consistent with the manufactures specifications.

To perform immunohistochemistry, sections of a tissue microarray (TMA), which has been previously described, were used^15^. Briefly, two core biopsies (diameter, 0.6 mm; length, 3–4 mm) from a morphologically representative area of interest of the paraffin ‘donor’ blocks were precisely arrayed into a new ‘recipient’ paraffin block using a stereomicroscope and the Beecher TMA instrument (Beecher Instruments, Sun Prairie, WI)^18^. From these (“recipient”) TMA paraffin blocks, 3.0 μm sections were freshly cut and used for further immunohistochemistry. The staining procedure was conducted according to the manufacturer’s instructions on an automated staining system (Leica Bond-III, Leica Biosystems, Wetzlar Germany). Briefly for PD-L1: Pretreatment Buffer H1, rabbit monoclonal anti PD-L1 (SP142) antibody (Spring Bioscience, Pleasanton, CA, USA)^19^. A tonsillectomy specimen served as positive control. A dilution of 1:100 resulted in a strong and distinct membranous staining pattern without unspecific background signal in positive controls (tonsil, Fig. 1). Staining intensity was analyzed using the percentage of positive cells (PP). Positivity for PD-L1 was defined as any unequivocal membranous staining of at least 1% of the tumor cells or the tumor infiltrating immune cells (TIIC)^1,20^. Multiple core’s PP were averaged and rounded to the nearest whole number. Two different authors (K.I. and D.V.), both blinded for the patient’s data, analyzed the percentage of positive tumor cells and TIIC.

### Statistics

Data was analyzed using descriptive statistics. Analysis of variance (ANOVA) and chi-square tests were performed to analyze baseline characteristics. The relationship between the PD-L1 expression and the clinico-pathologic variables such as TNM classification or tumor grading (which were used in its non-dichotomized form) were correlated using cross tabulation and chi square test. DFS, DSS and OS were compared with Kaplan-Meier survival analyses and log rank tests. SPSS statistics (IBM Corp; Armonk, NY), version 24, was used to assist with statistical analysis. A p-value < 0.05 was considered statistically significant.

## Results

Retrospective chart review enabled identification of 167 patients with malignant salivary gland tumors and a mean age of 57.2 ± 18.9 years (mean ± standard deviation, range 8–95 years). Eighty-three patients were women (49.7%), 84 men (50.3%). Tumor localization was in the parotid gland in 118 cases (70.6%), in the submandibular gland in 18 cases (10.8%), in the sublingual gland in one case (0.6%) and in other/accessory salivary glands in 30 patients (18.0%). Overall mean estimated DFS was 121.2 months (95% CI 105.5–137.0 months), DSS 225.7 months (95% CI 208.5–242.9 months) and OS 174.0 months (95% CI 152.4–195.5 months). Patients were followed-up over a mean period of 68.9 months (95% confidence interval (CI) 60.2–77.7 months, range 0–271 months).

As expected the well-established prognostic factors TNM classification, tumor grading, perineural, vascular and lympho-vascular invasion had a highly significant impact on DFS, DSS and OS (all p < 0.018).

**Figure 2:**
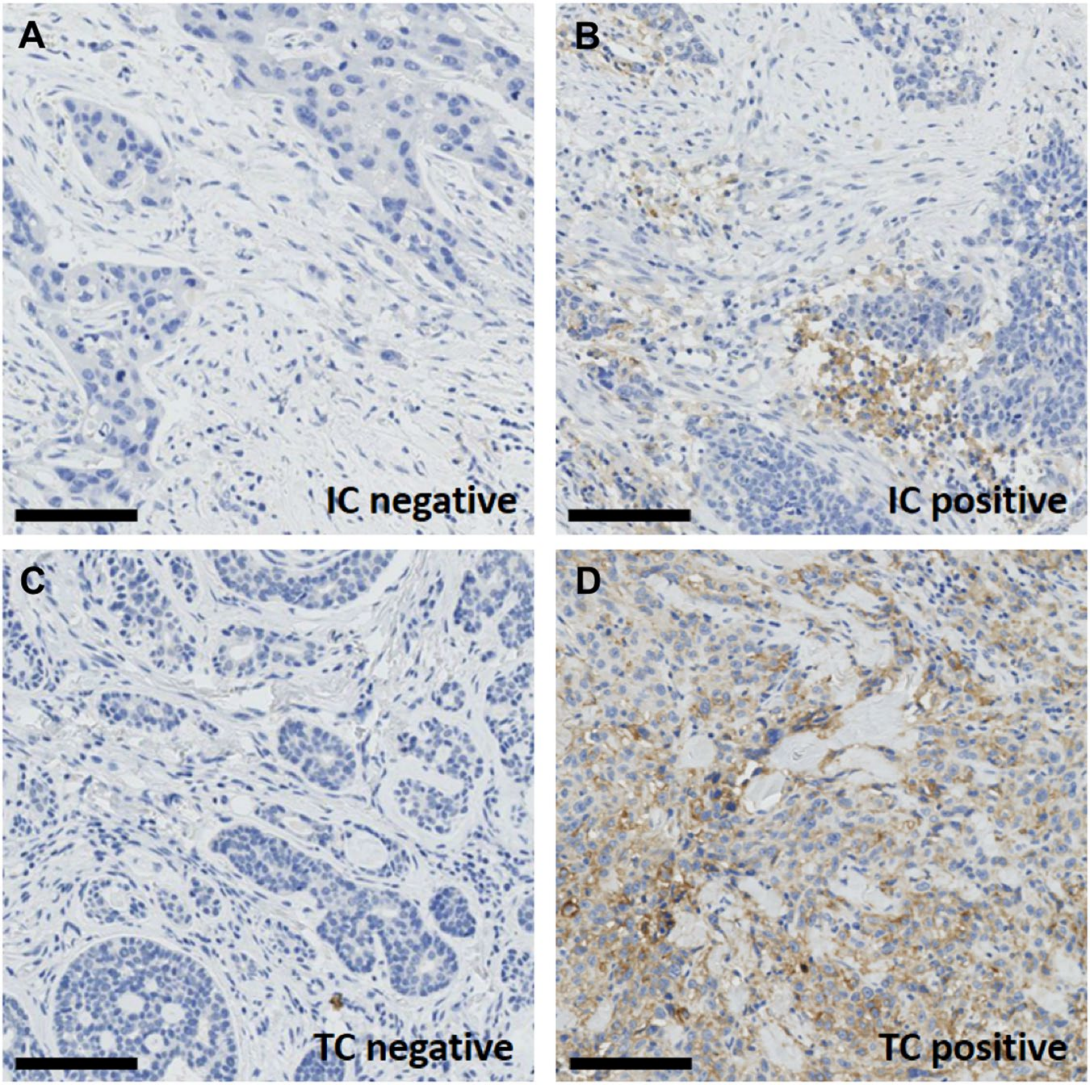
Representative immunohistochemical staining of salivary gland carcinoma demonstrating immunoreactivity in for PD-L1 in tumor-infiltrating immune cells ((**B**) corresponding H&E staining (**A**)) and in tumor cells ((**D**) corresponding H&E staining (**C**)). (**A**,**B** and **C**,**D**) are two different patients. Scale bar 100 μm, IC = immune cells, TC = tumor cells.

Baseline characteristics of the patients among PD-L1 positivity and negativity are depicted in Table 1. Of the 167 patients, 28 (16.8%) showed positivity of PD-L1 in tumor cells and 34 (20.4%) in TIIC (Fig. 2). Seventeen patients (10.2%) demonstrated positivity for PD-L1 in both in both tumor cells and TIIC (Table 2). Patients with PD-L1 positive tumor cells and both tumor cells and TIIC positive for PD-L1 showed a higher histologic tumor grade than their negative counterparts (p = 0.035 and p = 0.031, respectively), whereas there was no further correlation of PD-L1 expression with baseline characteristics. A trend towards a higher grading was present in TIIC PD-L1 positive tumors (p = 0.085). The four different groups of patients with PD-L1 positivity or negativity in tumor cells and/or TIIC (i.e. both tumor and TIIC positive, tumor positive/TIIC negative, tumor negative/TIIC positive, both tumor and TIIC negative) did not show any significant difference with regard to DFS, DSS or OS. Also, PD-L1 expression did not influence patient’s outcome if stratified by histologic tumor grading. The extent of immune infiltrate in relation to PD-L1 positivity is given in Table 3. In patients with PD-L1 positive TIIC, extent of immune infiltrate was significantly higher (p < 0.001).

**Table 1:** Characteristics of patients of with salivary gland carcinomas (n = 167) with respect to PD-L1 expression in tumor cells and tumor-infiltrating immune cells.

	*PD*-*L1 in tumor cells*			*PD*-*L1 in tumor*-*infiltrating immune cells*			*PD*-*L1 in tumor and tumor*-*infiltrating immune cells*		
	*positive* (*n* = *28*, *16*.*8*%)	*negative* (*n* = *139*, *83*.*2*%)	*p value*	*positive* (*n* = *34*, *20*.*4*%)	*negative* (*n* = *133*, *79*.*6*%)	*p value*	*positive* (*n* = *17*,*10*.*2*%)	*negative* (*n* = *150*, *89*.*8*%)	*p value*
*Age*	61.5 ± 22.7 years	56.4 ± 18.1 years	0.269	63.1 ± 20.6 years	55.7 ± 18.3 years	0.063	64.5 ± 24.7 years	56.4 ± 18.1 years	0.208
*Gender* (*F*/*M*)	15/13 (53.6/46.4%)	68/71 (48.9/51.1%)	0.653	16/18 (47.1/52.9%)	67/66 (50.4/49.6%)	0.730	9/8 (52.9/47.1%)	74/76 (49.3/51.7%)	0.804
*T classification*			0.178			0.714			0.376
*T1*	3 (10.7%)	32 (23.0%)		7 (20.6%)	28 (21.0%)		2 (11.8%)	33 (22.0%)	
*T2*	12 (42.9%)	29 (20.9%)		12 (35.3%)	29 (21.8%)		8 (47.1%)	33 (22.0%)	
*T3*	8 (28.6%)	33 (23.7%)		11 (32.4%)	30 (22.6%)		4 (23.5%)	37 (24.7%)	
*T4a*	3 (10.7%)	17 (12.2%)		3 (8.8%)	17 (12.8%)		3 (17.6%)	17 (11.3%)	
*T4b*	0	3 (2.2%)		1 (2.9%)	2 (1.5%)		0	3 (2.0%)	
*Tx*	2 (7.1%)	25 (18.0%)		0	27 (20.3%)		0	27 (18.0%)	
*N classification*			0.894			0.723			0.681
*N0*	11 (39.3%)	53 (38.1%)		14 (41.2%)	50 (37.6%)		6 (35.3%)	58 (38.6%)	
*N1*	1 (3.6%)	6 (4.3%)		2 (5.9%)	5 (3.8%)		1 (5.9%)	6 (4.0%)	
*N2a*	2 (7.1%)	6 (4.3%)		3 (8.9%)	5 (3.8%)		1 (5.9%)	7 (4.7%)	
*N2b*	6 (21.4%)	21 (15.2%)		8 (23.5%)	19 (14.2%)		5 (29.4%)	22 (14.7%)	
*Nx*	8 (28.6%)	53 (38.1%)		7 (20.5%)	54 (40.6%)		4 (23.5%)	57 (38.0%)	
*M classification*			0.073			0.904			0.543
*M0*	18 (64.3%)	111 (79.9%)		26 (76.5%)	103 (77.4%)		12 (70.6%)	117 (78.0%)	
*M1*	10 (35.7%)	28 (20.1%)		8 (23.5%)	30 (22.6%)		5 (29.4%)	33 (22.0%)	
*Mx*	0	0		0	0		0	0	
*Grading*			0.035*			0.058			0.031*
*G1*	6 (21.4%)	56 (40.3%)		11 (32.4%)	51 (38.3%)		2 (11.8%)	60 (40.0%)	
*G2*	4 (14.3%)	29 (20.9%)		3 (8.8%)	30 (22.6%)		3 (17.6%)	30 (20.0%)	
*G3*	18 (64.3%)	52 (37.4%)		20 (58.8%)	50 (37.6%)		12 (70.6%)	58 (38.7%)	
*Gx*	0	2 (1.4%)		0	2 (1.5%)		0	2 (1.3%)	
*Perineural invasvion*			0.096			0.054			0.305
*Pn0*	18 (64.3%)	63 (45.3%)		22 (64.7%)	59 (44.3%)		11 (64.7%)	70 (46.7%)	
*Pn1*	10 (35.7%)	71 (51.1%)		12 (35.3%)	69 (51.9%)		6 (25.3%)	75 (50.0%)	
*Pnx*	0	5 (3.6%)		0	5 (3.8%)		0	5 (3.3%)	
*Lymphatic vessel invasion*			0.515			0.230			0.404
*L0*	19 (67.9%)	99 (71.2%)		22 (64.7%)	96 (72.2%)		11 (64.7%)	107 (71.4%)	
*L1*	9 (32.1%)	35 (25.2%)		12 (35.3%)	32 (24.0%)		6 (35.3%)	38 (25.3%)	
*Lx*	0	5 (3.6%)		0	5 (3.8%)		0	5 (3.3%)	
*Blood vessel invasion*			0.056			0.891			1.000
*V0*	22 (78.6%)	122 (87.8%)		30 (88.2%)	114 (85.7%)		15 (88.2%)	129 (86.0%)	
*V1*	6 (21.4%)	12 (8.6%)		4 (11.8%)	14 (10.5%)		2 (11.8%)	16 (10.7%)	
*Vx*	0	5 (3.6%)		0	5 (3.8%)		0	5 (3.3%)	

Chi-square test: A p-value < 0.05 is considered significant.

**Table 2:** PD-L1 positivity in tumor cells versus tumor-infiltrating immune cells of different salivary gland carcinomas (n = 167).

		*Tumor cells*	
		*PD*-*L1 negative* (*n* = *139*)	*PD*-*L1 positive* (*n* = *28*)
*Tumor*-*infiltrating immune cells*	*PD*-*L1 negative* (*n* = *133*)	122 (73.0%)	11 (6.6%)
	*PD*-*L1 positive* (*n* = *34*)	17 (10.2%)	17 (10.2%)

**Table 3:** Extent of immune infiltrate versus PD-L1 positivity in tumor cells and tumor-infiltrating immune cells of different salivary gland carcinomas (n = 167).

		*PD*-*L1*		
		*Tu*+/*TIIC*+ (*n* = *17*)	*Tu*+/*TIIC*− (*n* = *11*)	*Tu*−/*TIIC*+ (*n* = *17*)
*Intensity of immune infiltrate*	*0* (*n* = *6*)	0	6 (54,5%)	0
	+ (*n* = *14*)	2 (11.8%)	3 (27.3%)	9 (53.0%)
	++ (*n* = *19*)	12 (70.6%)	2 (18.2%)	5 (29.4%)
	+++ (*n* = *6*)	3 (17..6%)	0	3 (17.6%)

**Figure 3:**
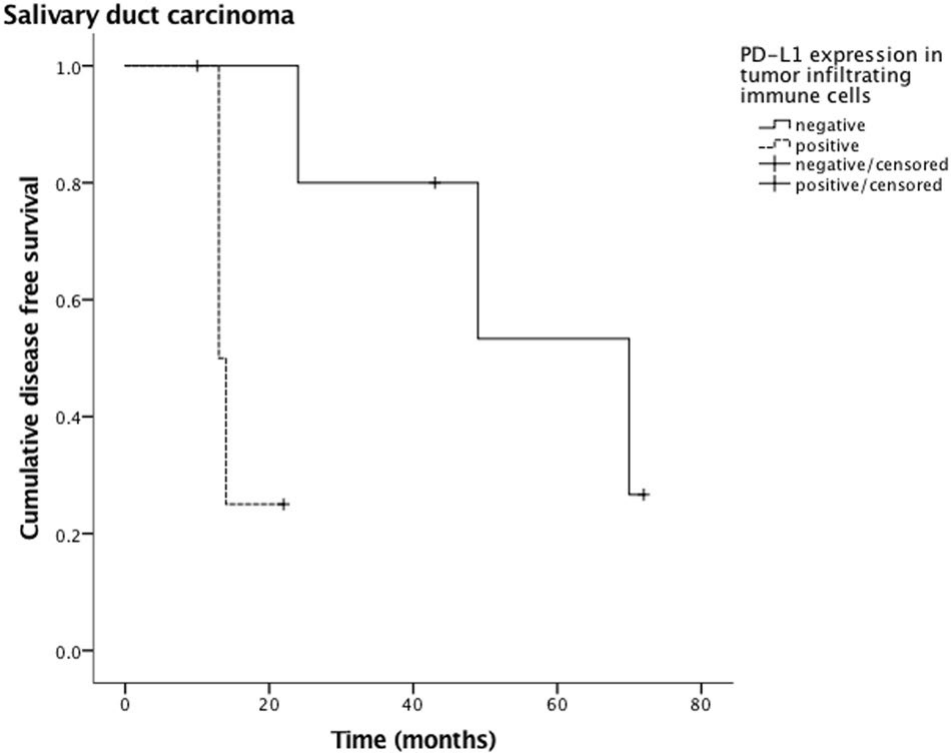
Disease-free survival of tumor infiltrating immune cell (TIIC) PD-L1 positive and negative patients with salivary duct carcinoma. The statistical difference is significant (p = 0.022).

**Figure 4:**
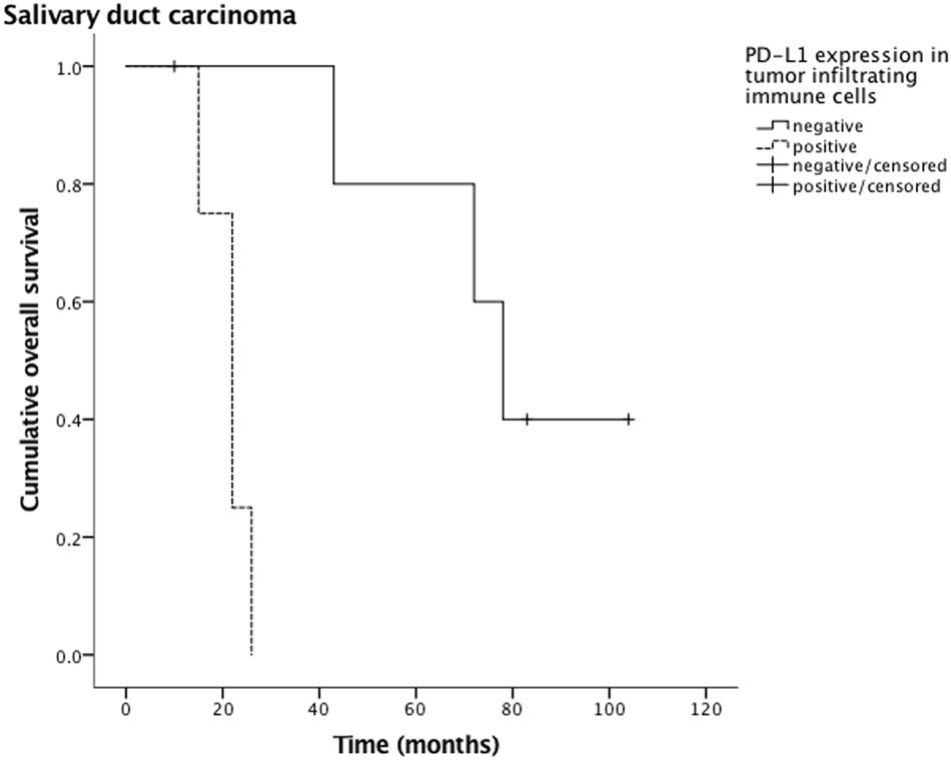
Overall survival of tumor infiltrating immune cell (TIIC) PD-L1 positive and negative patients with salivary duct carcinoma. The statistical difference is significant (p = 0.003).

**Figure 5:**
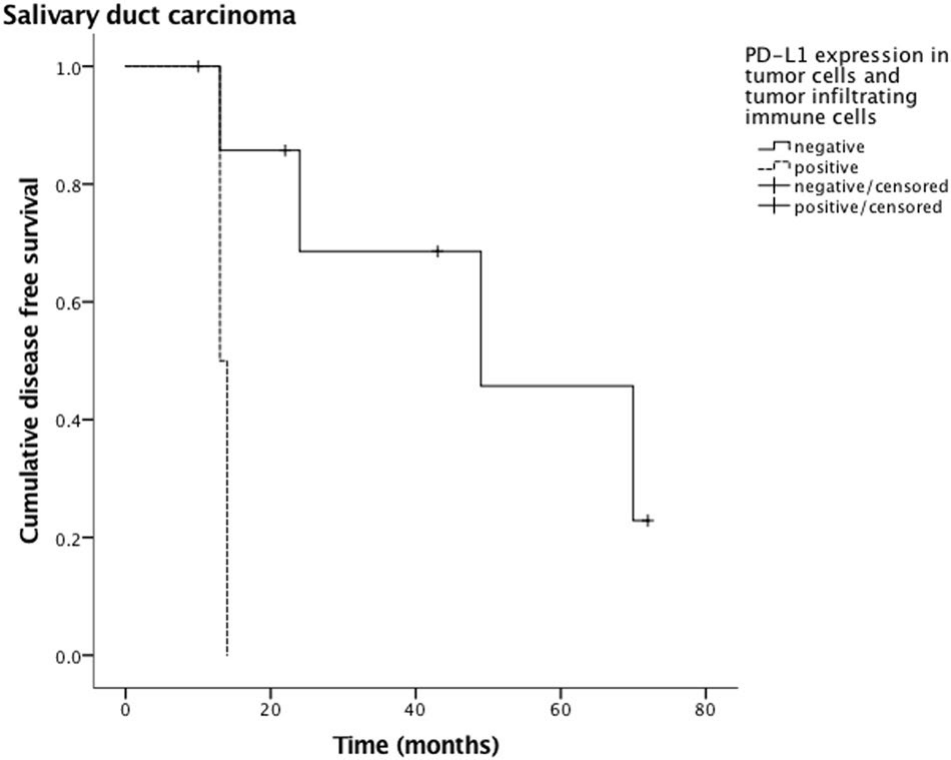
Disease-free survival of both PD-L1 positive salivary duct carcinoma cells and TIIC. The statistical difference is significant (p = 0.030).

PD-L1 expression in tumor cells and TIIC of to the various tumor entities are depicted in Table 4. Outcome analysis with regard to tumor subtype showed a worse DFS (Fig. 3) and OS (Fig. 4) in patients with salivary duct carcinoma and PD-L1 expressing TIIC (p = 0.022 and p = 0.003, respectively). This was also true with regard to DFS in patients with both SDC cells and TIIC being positive for PD-L1 (p = 0.030, Fig. 5). Due to the small size of the sub-cohort of patients with salivary duct carcinoma (n = 10), a multivariate analysis was not performed. All other subtypes of SGC did not show a relation between PD-L1 expression in tumor cells and/or TIIC and outcome.

**Table 4:** PD-L1 positivity in tumor cells and tumor-infiltrating immune cells of different salivary gland carcinomas (n = 167).

	*PD*-*L1 positivity in*	
	*tumor cells*	*tumor*-*infiltrating immune cells*
*Mucoepidermoid carcinoma* (*n* = *36*)	9 (25%)	13 (36%)
*Adenoic cystic carcinoma* (*n* = *36*)	3 (8%)	1 (3%)
*Acinic cell carcinoma* (*n* = *30*)	4 (13%)	6 (20%)
*Adenocarcinoma NOS* (*n* = *12*)	2 (17%)	4 (33%)
*Epithelial*-*myoepithelial carcinoma* (*n* = *11*)	0	2 (2%)
*Salivary duct carcinoma* (*n* = *10*)	3 (30%)	4 (40%)
*Carcinoma ex pleomorphic adenoma* (*n* = *10*)	1 (10%)	0
*Polymorphous low grade adenocarcinoma* (*n* = *7*)	1 (14%)	0
*Basal cell adenocarcinoma* (*n* = *3*)	1 (33%)	0
*Squamous cell carcinoma* (*n* = *2*)	1 (50%)	1 (50%)
*Small cell carcinoma* (*n* = *2*)	1 (50%)	1 (50%)
*Large cell carcinoma* (*n* = *1*)	1 (100%)	1 (100%)
*Lymphoepithelial carcinoma* (*n* = *1*)	1 (100%)	1 (100%)
*All carcinomas* (*n* = *167*)	28 (17%)	34 (20%)

Patients with oncocytic carcinoma (n = 2), myoepithelial carcinoma (n = 2), cystadenocarcinoma (n = 1) and carcinosarcoma (n = 1) did not show any PD-L1 positivity in either tumor cells or tumor-infiltrating immune cells.

## Discussion

Our retrospective analysis of PD-L1 expression in salivary gland carcinoma cells and in its tumor infiltrating immune cells reports the following major findings:

- Expression of PD-L1 (by means ≥1% of the cells with PD-L1 positivity) was present in the salivary gland carcinoma cells of 17% and in the TIIC of 20% of the patients. Ten percent of the patients demonstrated PD-L1 positivity for both tumor cells and TIIC.
- PD-L1 expression in tumor cells and both tumor cells and TIIC was related to a higher tumor grading (p = 0.035 and p = 0.030, respectively). A trend towards higher grading was also seen for PD-L1 expression in TIICs (p = 0.058).
- Patients with salivary duct carcinomas and PD-L1 expressing TIICs showed a significantly worse DFS and OS than their PD-L1 negative counterparts (p = 0.022 and p = 0.003, respectively), those with both SDC cells and TIIC expressing PD-L1 a significantly worse DFS (p = 0.030).

There is scarce literature on the role of PD-L1 in salivary gland carcinomas and only few other studies analyzing prognostic relevance of PD-L1 expression in different types of salivary gland carcinomas^1,6,21–23^: Mukaigawa *et al*. used the same threshold of ≥1% for PD-L1 positivity and demonstrated PD-L1 expression in a slightly higher fraction (23%) of the patient’s tumor cells and a moderately lower rate (13%) of the patient’s tumor-infiltrating mononuclear cells^1^. Harada *et al*. used a threshold of ≥5% for PD-L1 positivity and detected PD-L1 expression in 51.1% of the patients with salivary gland carcinomas^22^. Other groups, which analyzed the immunoprofile of adenoid-cystic carcinoma, did not find any expression of PD-L1^6^ or PD-1 positive SGC infiltrating T cells^23^. In our data, PD-L1 positivity of TIIC was furthermore associated with more extensive immune infiltrate. Consistent with the findings of Mukaigawa *et al*.^1^, PD-L1 expression in tumor cells and TIIC was associated with higher histological grading of SGC. However, a correlation with AJCC/UICC stage as reported by Harada *et al*. for membranous PD-L1 positivity^22^ or a correlation with age, sex, tumor localization, T and N stage as found by Mukaigawa *et al*.^1^ could not be confirmed with our data.

There is an ongoing debate about the prognostic role of PD-L1 (i.e. the prognostic implication of PD-L1 expression apart from the treatment with PD-1/PD-L1 blockers) with both favorable and unfavorable outcomes being reported in various malignancies^24–28^. Most of these authors conclude a negative impact of PD-L1 on outcome, which seems, looking to the data of Mukaigawa *et al*.^1^ and Harada *et al*.^22^, also to be true for salivary gland carcinomas. This effect is explained by its role as an immunosuppressive molecule, which interacts with the PD-1 receptor and leads to tumor protection and immunotolerance^29–31^. Indeed, the link between PD-L1 expression in both tumor and immune cells can roughly be explained by interferon gamma, which is produced by the tumor infiltrating immune cells and is one of the molecular mechanisms inducing PD-L1 expression^25,32,33^. The prognostic relevance of PD-L1 in our study is limited to patients with salivary duct carcinomas, demonstrating an adverse effect on outcome in patient with PD-L1 positivity of TIIC.

Salivary duct carcinoma (SDC) is a relatively rare, highly aggressive subtype of SGC, which most likely arises from the ductal epithelium and accounts for about 1–3% of all salivary gland malignancies^5,34–36^. It has one of the worst outcomes within the group of SGC. Patients with SDC show rapid progression of disease and a high incidence of metastasis (distant metastatic disease in 40–70%), which leads to tumor-related death within 3 years in the majority of the patients^37–39^. Due to its very low incidence, literature is limited to small retrospective studies and the optimal treatment remains still unclear, especially given the fact that current adjuvant treatments do not have a detectable impact on survival and the effect of chemotherapies for unresectable or metastatic tumors remains minimal^1,5–8^.

Looking at the paucity of literature on SDC, it is difficult to discuss our findings by reviewing of the contemporary literature. To our knowledge, there is only one article considering PD-L1 receptor expression as a prognostic factor of SDC, which studied 67 patients with SDC or SDC ex pleomorphic adenoma. However, the authors Haderlein *et al*. failed to demonstrate a significant relationship between PD-L1 receptor expression and outcome of patients with SDC^21^. A more recent study by Sato *et al*. analyzed a small collective of 12 patients with SDC and found an unfavorable prognosis in patients with PD-L1 positive tumor cells^40^. Invasive ductal carcinoma of the breast, furthermore, resembles SDC and shows similar immunohistochemical staining patterns^37,41,42^. It is well known that PD-L1 expression in ductal carcinoma of the breast is correlated with high-risk features such as tumor grading and therefore could be an indicator of advanced stage and poor prognosis^30,43,44^. The adverse effect of PD-L1 expression in TIIC on outcome in the context of invasive ductal breast cancer can be explained by the following effects: An inhibition of T lymphocyte clonal expansion either by reverse signaling processes or by inhibition of other T lymphocytes by binding to its receptors (T cell-T cell interaction)^30,45,46^.

Our study has several limitations: It is a retrospective study on a robust number of SGCs, which consists of several different tumor entities. Consequently, the subgroups of the different entities are small. However, the statistical findings in the rare, but clinically relevant subgroup of SDC are significant. These findings merit evaluation in more detailed studies with larger numbers of SDC in a multicenter setting, which might be applicable to future studies on the usefulness of treatment with PD-1/PD-L1 inhibitors in SGC, or SDC in particular.
